# Introduction: Untold Legacies of the First World War in Britain

**DOI:** 10.1179/0729247314Z.00000000048

**Published:** 2015-06-21

**Authors:** Alison S. Fell, Jessica Meyer

**Affiliations:** ^a^ University of Leeds

**Keywords:** First World War, Britain, historiography, Legacies of War

## Abstract

The current centenary of the First World War provides an unrivalled opportunity to uncover some of the social legacies of the war. The four articles which make up this special issue each examine a different facet of the war’s impact on British society to explore an as yet untold story. The subjects investigated include logistics, the history of science, the social history of medicine and resistance to war. This article introduces the four which follow, locating them in the wider historiographic debates around the interface between warfare and societies engaged in war.

It is a truism that the First World War had a dramatic impact on twentieth-century British society. The expansion of the British electorate, the greater opportunities available to women, and an improvement of working-class standards of living, have all been defined as legacies of the war which continue to resonate powerfully today. However, there remain important ongoing historical debates over the extent to which the war acted as instigator, accelerator, or even brake on social, cultural, and economic change in British society. For instance, the discussions around changes to the British franchise, women’s place in society, and improvements in living standards and their relationship to the war are increasingly being qualified.^1^ Where the war was often portrayed, both at the time and since, as a watershed moment,^2^ historical studies have more recently emphasized continuities as much as radical transformations.^3^ The current centenary of the war years provides an unrivalled opportunity to re-examine some of these debates. This special issue aims, in particular, to uncover some of the lesser-known legacies of the war that, even a century later, have yet to be fully explored.

Recent trends in the scholarship of the social history of the First World War have included re-evaluations of the technological and medical advances made during the war,^4^ as well as new enquiries into the relationship between the war and philanthropy.^5^ A resurgent interest in the home front during the war has led to investigations of the place of food and drink in wartime society,^6^ as well as broader studies of life and morale on the home front.^7^ Such studies extend and complicate our understanding of the impacts of the war, demonstrating both the extent to which all aspects of British society were affected and the long-term legacies which could be left in the most unexpected places. Seth Koven’s analysis of the relationship between the treatment of disabled children and disabled ex-servicemen in Britain, for example, explores not only the two-way relationship between the two groups but also the legacies these relationships had for social perceptions of disability in the longer term.^8^ Similarly, Jeffrey Reznick’s work on wartime hospitals locates medical and architectural developments in wartime in both the history of medicine in wartime and theoretical developments in medical rehabilitation.^9^

The articles collected here seek to add to this body of scholarship through their exploration of four facets of the war. All four are drawn from projects associated with the Legacies of War research and engagement hub based at the University of Leeds. This project involves a team of researchers focusing on social, cultural, and medical histories of the war, often working from a European, comparative perspective. The members of the research team have been working not only alongside other university academics but also alongside museum professionals, community researchers, and cultural practitioners. They have thereby sought to extend both academic and public knowledge and understanding of the diverse legacies of the war in five key areas of study. A central goal has to been to explore less mined primary sources, and to collaborate with researchers working in different contexts, such as museums, schools and theatres, or different disciplines in order to bring new perspectives to bear on more well-worn interpretations. The four themes addressed here involve three of the hub’s key themes: science and technology, medicine and war, and resistance to warfare.

As with all scholarship, these articles seek to uncover original, previously untold stories. In two cases, Chris Phillips’s article on Francis Dent’s attempts to organize British military transportation at Bassin Loubet, and Dominic Berry’s exploration of the foundation and work of the Seed Testing Service (STS) during the war and in its immediate aftermath, the untold story is that of the event or institution itself. These are historical moments and organizations that have not, as yet, received significant scholarly attention, and it is only now that their stories are starting to be told and their legacies explored.^10^ In telling them, Phillips and Berry not only uncover the histories of their subjects, but also their legacies for civil–military relations and the history of agricultural science and its place in society.

Superficially, the subject matter of the other two articles, Jessica Meyer’s examination of the British Red Cross and the Friends’ Ambulance Unit (FAU), and Cyril Pearce and Helen Durham’s discussion of patterns of conscientious objection to war, are more familiar, with studies of both already in existence.^11^ Yet, by applying new methodological approaches in one case, and bringing the fruits of long-term quantitative research and analysis to bear on the subject in the other, the authors shed new light on seemingly familiar topics. In doing so, they are able to illuminate not only the topics themselves but also the social legacies they had for wartime voluntarism and resistance to war as a community stance, respectively.

What unites these four articles, moreover, is not simply the originality of topic or approach, but rather the fact that each, in its own way, expresses the complex interface between warfare and the society engaged in war. This proves to be an interdependent relationship: whereas, more typically, war is seen to be the active agent in the relationship, leaving its traces on the society in which it takes place, these articles also show that the existing structures, organizations, and modes of thinking in British society had a crucial impact upon the ways in which the First World War was fought, organized, understood, and responded to. Phillips’s article, dealing as it does explicitly with civil–military relations, provides the most obvious example of this two-way interplay between war and society, but the social importance of the questions raised is evident in the other three articles as well. Pearce’s uncovering of unsuspected pockets of resistance to war through his analysis of the conscientious objectors’ (CO) database provides important insight into the variations of attitude towards the war that existed across the country, as well as into the significance of pre-existing social and regional networks. This complicates our understanding of war enthusiasm at the local level, giving perspective to recent analyses of war enthusiasm at the national level.^12^ Conversely, Meyer’s article on medical voluntarism problematizes both readings of the FAU as a space of resistance to war and of medical voluntarism as a humanitarian endeavour. These moral nuances continue to inflect understandings of the role of voluntary medical caregiving in conflicts today. Finally, Berry’s analysis of the work of the STS demonstrates how scientific endeavour both drew on and informed contemporary debates about eugenics, the role of women in the workforce, and the place of disabled ex-servicemen in post-war society, to shape both the direction of the institution itself and the society in which it was located.

Read together, these four articles depict the relationship between the First World War as waged by Britain, and the society engaged in waging it, as a complex series of interactions between a large number of stakeholders. Many of the individuals discussed cannot be categorized simply as military or civilian, militaristic or resistant to war. All the actors and institutions discussed were negotiating their relationships within a society redefining itself in light of the total war. For some of them, this involved a new application of pre-war skills, experiences, or ideologies to a wartime context; for others, it involved a more radical re-thinking of pre-war norms. The compromises and accommodations that they made, or resisted making, to do so have left legacies for British society today, ranging from understandings about the role of medical aid in conflict to discussions of the roles of women in scientific laboratories. Nor is it only the legacies of the historic subjects that these articles demonstrate. The CO Register compiled by Pearce, now hosted on-line by the Imperial War Museum’s Lives of the First World War project,^13^ is a historic legacy in its own right as a source of information for future generations of social and cultural historians. In both exploring and creating legacies of the First World War in Britain, these articles show how much the untold legacies of the First World War still have to inform us about that much-discussed conflict.

## Notes on contributors

Alison S. Fell is Professor of French Cultural History at the University of Leeds. She has published widely on French and British women’s responses to the First World War. She is currently leading *Legacies of War*, a research and public engagement hub at the University of Leeds (https://arts.leeds.ac.uk/legaciesofwar)

Jessica Meyer is a University Academic Fellow in the School of History at the University of Leeds. Her current research on masculinity and medical care during the First World War examines the roles and status of non-commissioned servicemen in the Royal Army Medical Corps. She has published extensively on the history of masculinity, war disability and popular fiction. Her monograph on British soldiers’ masculine identity during the First World War, *Men of War: Masculinity and the First World War in Britain*, was published by Palgrave Macmillan in 2009.
